# The *XRCC1* Arg194Trp polymorphism was associated with the risk of head and neck squamous cell carcinoma development: Results from a systematic review and meta‐analysis

**DOI:** 10.1002/cnr2.1776

**Published:** 2022-12-27

**Authors:** Nooshin Mohtasham, Khadijeh Najafi‐Ghobadi, Hamid Abbaszadeh

**Affiliations:** ^1^ Oral and Maxillofacial Diseases Research Center Mashhad University of Medical Sciences Mashhad Iran; ^2^ Department of Biostatistics, School of Public Health Hamadan University of Medical Sciences Hamadan Iran; ^3^ Department of Oral and Maxillofacial Pathology, School of Dentistry Birjand University of Medical Sciences Birjand Iran

**Keywords:** genetic polymorphism, squamous cell carcinoma of head and neck, X‐ray repair cross complementing protein 1

## Abstract

**Background:**

The X‐ray repair cross complementing group 1 (*XRCC1*) is a DNA repair gene. Various studies have examined the association between *XRCC1* Arg194Trp polymorphism and head and neck squamous cell carcinoma (HNSCC) susceptibility with contradictory results. So, this systematic review and meta‐analysis aimed to assess whether variants of this polymorphism increase the HNSCC risk or not.

**Recent findings:**

Thirty three studies consisting of 14282 subjects (6012 cases and 8270 controls) were included in this meta‐analysis. Variants of XRCC1 Arg194Trp polymorphism were associated with increased HNSCC risk and the associations were significant based on heterozygous and dominant models (heterozygous model: OR = 1.182, 95%CI = 1.015–1.377, P = 0.032; homozygous model: OR = 1.274, 95%CI = 0.940–1.727, P = 0.119; dominant model: OR = 1.194, 95%CI = 1.027–1.388, P = 0.021; recessive model: OR = 1.181, 95%CI = 0.885–1.576, P = 0.119). There were significant associations between variants of this polymorphism and HNSCC risk based on Asian ethnicity under dominant model, hospital control source under different genetic models, PCR‐RFLP genotyping method under dominant model and oral cavity tumor site under heterozygous and dominant models.

**Objective:**

The X‐ray repair cross complementing group 1 (*XRCC1*) is a DNA repair gene. Various studies have examined the association between *XRCC1* Arg194Trp polymorphism and head and neck squamous cell carcinoma (HNSCC) susceptibility with contradictory results. So, this systematic review and meta‐analysis aimed to assess whether variants of this polymorphism increase the HNSCC risk or not.

**Methods:**

A systematic search of the literatures published till April 2022 was conducted using Google Scholar, Scopus, PubMed, Web of Science, Cochrane Library and Embase databases. The heterogeneity was assessed with the I‐Square statistic. A random effects model or fixed effects model was used to analyze the data. Data were reported by odds ratio (OR) and 95% confidence interval (CI). The *p* value was considered significant if *p* < .05.

**Results:**

Thirty three studies consisting of 14 282 subjects (6012 cases and 8270 controls) were included in this meta‐analysis. Variants of *XRCC1* Arg194Trp polymorphism were associated with increased HNSCC risk and the associations were significant based on heterozygous and dominant models (heterozygous model: OR = 1.182, 95%CI = 1.015–1.377, *p* = .032; homozygous model: OR = 1.274, 95%CI = 0.940–1.727, *p* = .119; dominant model: OR = 1.194, 95%CI = 1.027–1.388, *p* = .021; recessive model: OR = 1.181, 95%CI = 0.885–1.576, *p* = .119). There were significant associations between variants of this polymorphism and HNSCC risk based on Asian ethnicity under dominant model, hospital control source under different genetic models, PCR‐RFLP genotyping method under dominant model and oral cavity tumor site under heterozygous and dominant models.

**Conclusion:**

Variants of *XRCC1* Arg194Trp polymorphism were significantly associated with increased risk of HNSCC development based on heterozygous and dominant genetic models.

## 1 INTRODUCTION

Head and neck squamous cell carcinomas (HNSCCs) consist of the SCCs of the oral cavity, pharynx, and larynx. HNSCC is the fifth most common cancer worldwide, with severe morbidity and mortality and a poor overall survival rate.^1^ Development of HNSCC is a multifactorial process. Varied risk factors have been involved in the carcinogenesis process. Tobacco use and alcohol drinking are among the major risk factors for HNSCC.^1^, ^2^, ^3^ These carcinogens can cause DNA damage; this may induce apoptosis or may lead to uncontrolled cell proliferation and consequently cancer.^1^, ^2^

The DNA repair genes play an important role in maintaining the genomic integrity by repairing the DNA. So, the mutation of DNA repair genes may increase the risk of HNSCC.^3^ The X‐ray repair cross‐complementing group 1 (*XRCC1*) gene is involved in the base excision repair (BER) pathway and protects DNA from the harmful effects of carcinogens. *XRCC1* protein plays a significant role in repairing single‐stranded DNA fractures.^3^, ^4^ The *XRCC* genes have important roles in different DNA repair processes which prevent genomic instability. Genetic polymorphisms in DNA repair genes such as *XRCC1* may change the DNA repair capacity which subsequently has impacts on cancer susceptibility.^1^, ^2^, ^3^, ^4^

Several studies have assessed the association between *XRCC1* polymorphisms and cancer risk and prognosis; they have shown that individual susceptibility to cancer is different because of *XRCC1* gene polymorphisms in lung, breast, stomach, esophageal and nasopharyngeal cancers.^4^ It has been hypothesized that *XRCC1* gene polymorphisms may alter HNSCC risk. However, the results of several studies that have examined this hypothesis are contradictory.^1^, ^2^, ^4^

One of the most common single nucleotide polymorphisms in the *XRCC1* gene is Arg194Trp (C to T transition at exon 6 which results in arginine [Arg] to tryptophan [Trp] amino acid change).^4^

So, this systematic review and meta‐analysis aimed to assess whether variants of *XRCC1* Arg194Trp polymorphism increase the risk of development of HNSCC or not.

## 2 MATERIALS AND METHODS

This meta‐analysis has been registered in PROSPERO (ID: CRD42022336289).

### 2.1 Search strategy

**FIGURE 1:**
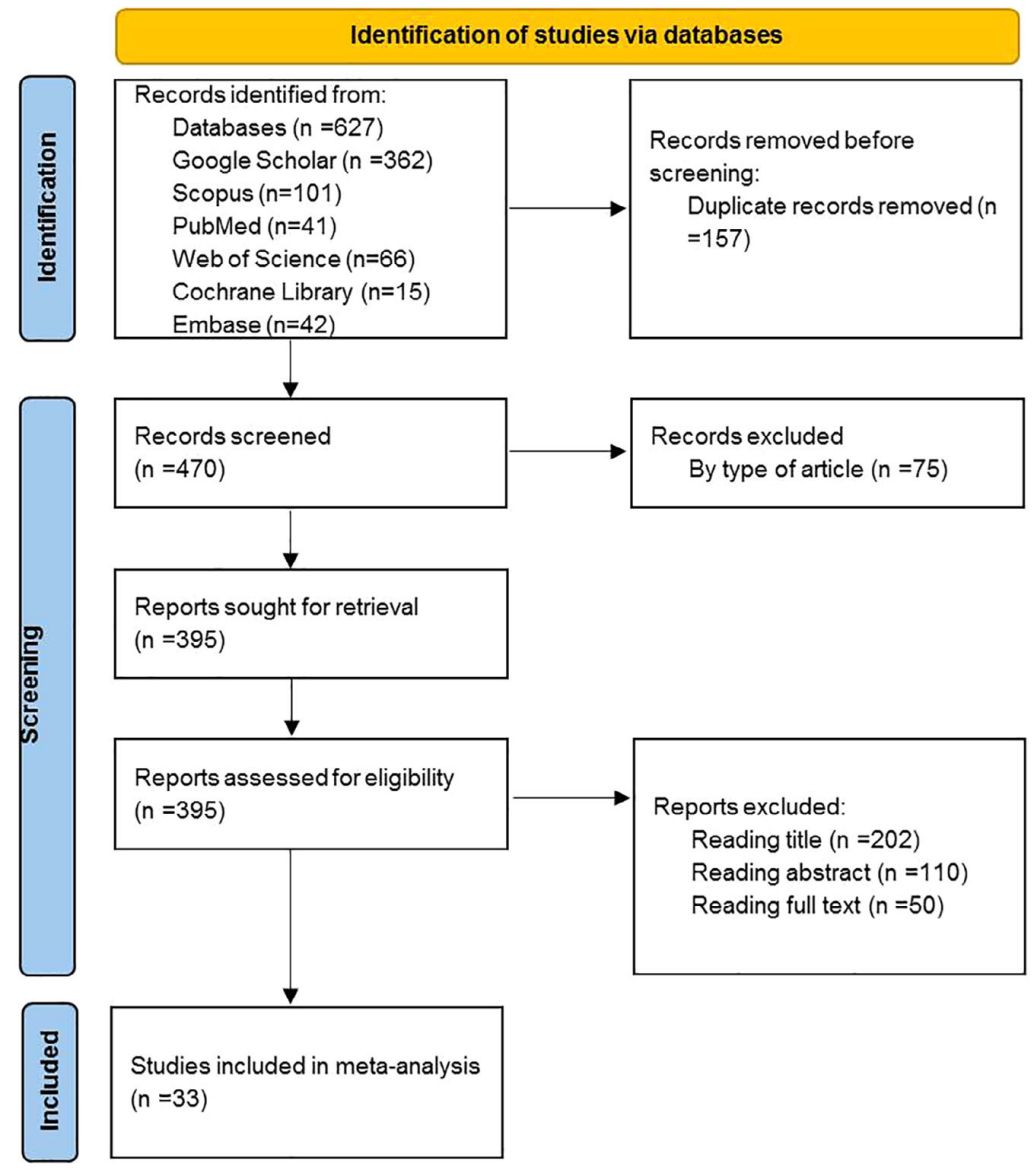
PRISMA flow diagram representing process of identification of studies through databases

We used Preferred Reporting Items for Systematic Reviews and Meta‐Analyses (PRISMA) guidelines for the systematic review reporting. PRISMA flow diagram was used for systematic identification and selection of studies (Figure 1).

The search strategy was based on the research question (PICO):

Do variants of *XRCC1* Arg194Trp polymorphism increase the risk of development of HNSCC?

P = Population/Patient: HNSCC patients; I = Intervention: presence of gene polymorphism; C = comparator: individuals without polymorphism; O = outcome: risk or susceptibility.

The search process was based on the MeSH terms as keywords using the following algorithm:

(“Genetic polymorphism” OR “single nucleotide polymorphism” OR “genetic variation”) AND (“X‐ray repair cross complementing protein 1” OR “DNA repair” OR “rs 1 799 782”) AND (“disease susceptibility” OR “risk”) AND (“head and neck neoplasms” OR “squamous cell carcinoma of head and neck” OR “mouth neoplasms” OR “nasopharyngeal carcinoma” OR “laryngeal neoplasms” OR “pharyngeal neoplasms”)

### 2.2 Searched sources

The sources searched included Google Scholar, Scopus, PubMed, Web of Science, Cochrane Library, and Embase databases.

### 2.3 Inclusion and exclusion criteria

This meta‐analysis included all studies that had been published up to April 2022 regarding the association of *XRCC1* Arg194Trp polymorphism with HNSCC risk and contained the required information including the frequency of different alleles and genotypes of the *XRCC1* Arg194Trp polymorphism. There was no restriction on the language of the article.

In cases where the full text of the article could not be accessed and the required information was not available in the abstract of the article, that study was excluded from the meta‐analysis. Case report, review and letters to the editor articles were excluded from the meta‐analysis. Studies that did not have the required quality after risk of quality assessment were also excluded (e.g., score below 60%).

### 2.4 Data extraction

The systematic search and article selection was done by two independent researchers. In cases where there was disagreement between the two researchers, the disagreement was resolved by consensus, or in the absence of consensus, the final decision was made by referring to another third researcher.

All initial searched articles were first screened by their titles; articles with unrelated titles to the aim of this meta‐analysis as well as articles with duplicate titles were excluded. In the next step, the remained searched articles were screened by their abstracts. At final step, the screening process was completed by assessing the full‐texts of articles. EndNote X7 software was used to manage the data.

A designed form in Microsoft Excel 2016 software was used as a data collection tool. This form contained the following information: author, publication year, country, ethnicity, control source, tumor site, genotyping methods, sample size (case/control), frequency of Arg allele (case/control), frequency of Trp allele (case/control), frequency of Arg/Arg genotype (case/control), frequency of Arg/Trp genotype (case/control), frequency of Trp/Trp genotype (case/control), Hardy–Weinberg Equilibrium (HWE) *p* value, and quality score.

### 2.5 Risk of bias (quality) assessment

Quality assessment was done through evaluation of the following items: quality of methodology, accuracy of study, and external validity.

In this meta‐analysis, the Joanna Briggs Institute (JBI) checklist for case control studies^5^ was used to assess the quality of the studies in terms of clear criteria for inclusion, detail description of study subject and setting, reliability and validity of study tools, used standard criteria or objective, identify cofounding factor, strategy dealing with cofounders, outcome measured in a valid way, appropriate statistical analysis. Articles with a score below than 60% according to this checklist would be excluded.

Risk of bias (quality) assessment was done by two independent researchers. The disagreement between these two researchers was being resolved by consensus or referral to a third reviewer.

### 2.6 Data analyses

Publication bias was assessed using funnel plot and Begg's test (*p* value <.05 was considered as having publication bias). *I* ^2^ statistic (significance level ≥25%) was used to identify statistical heterogeneity. If there was heterogeneity, the random effects model was used to analyze the data; otherwise the fixed effects model was used. Subgroup analysis was used to deal with the heterogeneity. There was no adjustment for multiple comparisons. The *p* value was considered significant if *p* < .05.

All analyses were performed by Stata 12 statistical software and Comprehensive Meta‐Analysis Software.

## 3 RESULTS

Of initial 627 searched articles, 33 studies were eligible to include in this meta‐analysis. All of these studies had a quality score of higher than 60%. These 33 studies consisted of 14 282 subjects including 6012 cases and 8270 controls. The sex distribution ranged from 63.5% to 100% for male HNSCC patients and 0%–36.5% for female HNSCC patients. The sex distribution in control group ranged from 49.3% to 100% for male individuals and 0%–50.7% for female individuals. The age distribution ranged from 12 to 89 years old for HNSCC patients. The age distribution in control individuals ranged from 20 to 84 years old.

Table 1 summarizes the characteristics of the studies included in this meta‐analysis.

**TABLE 1:** Characteristics of the studies included in this meta‐analysis

First Author, year	Ethnicity	Control source	Tumor site	Genotyping methods	Sample size (case/control)	Sex (male/female)	Mean age (years)
Sturgis, 1999^6^	Caucasian	Hospital	Oral cavity, larynx, pharynx	PCR‐RFLP	203/424	Case: 137/66 Control: 269/155	Case: 59.8 Control: 60.1
Olshan, 2002^7^	Caucasian	Hospital	Oral cavity, larynx, pharynx	PCR‐RFLP	98/161	Case: 71/27 Control: 90/71	Case: 61.8 Control: 57.6
Varzim, 2003^8^	Caucasian	Healthy	Larynx	PCR‐RFLP	88/178	Case: 83/5 Control: 128/50	Case: 62.8 Control: 43.02
Tae, 2004^9^	Asian	Hospital	Oral cavity, larynx, pharynx	Sequence	129/157	NS	NS
Demokan, 2005^10^	Caucasian	Healthy	Oral cavity, larynx, pharynx	PCR‐RFLP	95/98	Case: 83/12 Control: 51/47	Case: 59.6 Control: 47.2
Majumder, 2005^11^	Asian	Hospital	Oral cavity	PCR‐RFLP	310/348	Case: 197/113 Control: 265/83	Case: 55 Control: 50.4
Rydzanicz, 2005^12^	Caucasian	Healthy	Oral cavity, larynx, pharynx	PCR‐RFLP	182/143	Case: 178/4 Control: 143/0	Case: 61.2 Control: 53.1
Gajecka, 2005^13^	Caucasian	Healthy	Larynx	PCR‐RFLP	293/319	Case: 293/0 Control: 319/0	NS
Kietthubthew, 2006^14^	Asian	Healthy	Oral cavity	PCR‐RFLP	106/164	Case: 77/29 Control: 91/73	Case: 67.1 Control: 68.4
Matullo, 2006^15^	Caucasian	Healthy	Oral cavity, larynx, pharynx	Taqman	82/1094	NS	NS
Cao, 2006^16^	Asian	Healthy	Pharynx	PCR‐RFLP	425/501	Case: 339/123 Control: 252/259	Case: 45.9 Control: 45.7
Ramachandran, 2006^17^	Asian	Hospital	Oral cavity	PCR‐RFLP	110/110	NS	NS
Majumder, 2007^18^	Asian	Hospital	Oral cavity	PCR‐RFLP	309/387	Case: 198/112 Control: 302/87	Case: 55 Control: 49
Yang, 2007^19^	Asian	Healthy	Pharynx	PCR‐RFLP	153/168	Case: 110/43 Control: 118/50	Case: 48.7 Control: 47.9
Yen, 2008^20^	Asian	Hospital	Oral cavity	PCR‐RFLP	103/98	Case: 100/3 Control: 49/49	Case: 53.6 Control: 40.5
Harth, 2008^21^	Caucasian	Hospital	Oral cavity, larynx, pharynx	PCR‐RFLP	310/300	Case: 250/60 Control: 176/124	Case: 59.7 Control: 47.2
Csejtei, 2009^22^	Caucasian	Healthy	Oral cavity, larynx, pharynx	PCR‐RFLP	108/102	Case: 97/11 Control: NS	Case: 56.7 Control: NS
Kowalski, 2009^23^	Caucasian	Healthy	Oral cavity, larynx, pharynx	PCR‐RFLP	92/124	Case: 50/42 Control: 63/61	Case: 48.7 Control: 44.47
Applebaum, 2009^24^	Caucasian	Healthy	Oral cavity, larynx, pharynx	PCR‐RFLP	483/547	Case: 359/124 Control: 401/146	Case: 59.5 Control: 61
Gugatschka, 2011^25^	Caucasian	Healthy	Oral cavity, larynx, pharynx	Taqman	168/463	Case: 148/20 Control: 234/229	Case: 65 Control: 58
Laantri, 2011^26^	African	Hospital	Pharynx	Taqman	512/477	NS	NS
Kumar, 2012^27^	Asian	Healthy	Oral cavity, larynx, pharynx	PCR‐RFLP	278/278	Case: 278/0 Control: 278/0	Case: 50 Control: 52
Dos Reis, 2013^28^	Mixed	Healthy	Oral cavity	PCR‐RFLP	150/150	Case: 122/28 Control: 122/28	Case: 57.52 Control: 57.27
Curioni, 2013^29^	Mixed	Hospital	Oral cavity	PCR‐RFLP	92/244	Case: 81/11 Control: 225/19	Case: 53 Control: 53.6
Zhu, 2014^30^	Asian	Healthy	Pharynx	PCR‐RFLP	87/94	NS	NS
Mutlu, 2015^31^	Caucasian	Healthy	Oral cavity, larynx, pharynx	PCR‐RFLP	55/69	NS	NS
Yang, 2015^32^	Asian	Hospital	Oral cavity	PCR‐RFLP	103/98	Case: 100/3 Control: 49/49	NS
Costa, 2016^33^	Mixed	Hospital	pharynx	PCR‐RFLP	200/200	Case: 183/17 Control: NS	Case: 57 Control: 53
Alimu, 2018^34^	Asian	Healthy	Larynx	PCR‐RFLP	58/116	Case: 49/9 Control: NS	Case: 62 Control: 58
Borkotoky, 2020^35^	Asian	Healthy	Oral cavity	PCR‐RFLP	152/190	Case: 109/43 Control: NS	Case: 52.96 Control: NS
Kabzinski, 2021^36^	Caucasian	Healthy	Oral cavity	Taqman	353/343	Case: 204/149 Control: NS	Case: 63 Control: NS
Seifi, 2022^37^	Asian	Healthy	Oral cavity	PCR‐RFLP	50/59	Case: 36/14 Control: 35/24	Case: 62.04 Control: 54.15
Tata, 2022^38^	Asian	Hospital	Oral cavity	PCR‐RFLP	75/75	Case: 51/24 Control: 54/21	Case: 54.88 Control: NS

Abbreviation: NS, not specified.

### 3.1 Publication bias

Results of Begg's test showed no publication bias except for subgroup analyses in Asian ethnicity under allelic genetic model (*p* value = .038), Taqman genotyping method under heterozygous and dominant genetic models (*p* values = .042) and oral cavity tumor site under allelic genetic model (*p* value = .016).

### 3.2 Meta‐analysis results

Table 2 summarizes the results of meta‐analysis on the association of *XRCC1* Arg194Trp polymorphism with HNSCC risk in different subgroups.

**TABLE 2:** The association of *XRCC1* Arg194Trp polymorphism with HNSCC risk in different subgroups

Statistic Subgroup			OR (95%CI)	*p* Value	Heterogeneity (*I* ^2^ Statistic [%])
Genetic model	Allelic		0.86 (0.75–0.98)	.029	64.5
	Heterozygous		1.182 (1.015–1.377)	.032	58.7
	Homozygous		1.274 (0.940–1.727)	.119	36.2
	Dominant		1.194(1.027–1.388)	.021	61.2
	Recessive		1.181 (0.885–1.576)	.258	34.2
Ethnicity	Asian	Allelic	.764 (.613–.952)	.016	79
		Heterozygous	1.256(0.973–1.620)	.080	72.1
		Homozygous	1.447 (0.908–2.307)	.121	65.3
		Dominant	1.329 (1.033–1.710)	.027	75.6
		Recessive	1.350 (0.897–2.032)	.150	56.9
	Caucasian	Allelic	1.040 (0.916–1.180)	.548	0
		Heterozygous	1.038 (0.851–1.267)	.713	31
		Homozygous	0.985 (0.674–1.438)	.936	0
		Dominant	1.033(0.889–1.200)	.670	8.7
		Recessive	0.737 (0.529–1.026)	.071	0
	Mixed	Allelic	0.748 (0.413–1.354)	.337	67.8
		Heterozygous	1.589 (0.927–2.721)	.092	54.3
		Homozygous	0.650 (0.195–2.167)	.483	0
		Dominant	1.479 (0.833–2.627)	.181	52.7
		Recessive	0.615 (0.184–2.055)	.429	0
Control source	Healthy	Allelic	0.988 (0.841–1.160)	.880	59.2
		Heterozygous	1.079 (0.887–1.313)	.446	57.5
		Homozygous	1.017 (0.709–1.458)	.927	31
		Dominant	1.065 (0.874–1.296)	.533	61.2
		Recessive	0.811 (0.650–1.013)	.065	16.3
	Hospital	Allelic	0.715 (0.595–0.860)	<.001	51.7
		Heterozygous	1.350 (1.075–1.697)	.010	54.2
		Homozygous	1.869 (1.306–2.674)	.001	10
		Dominant	1.401 (1.155–1.700)	.001	44.8
		Recessive	1.781 (1.253–2.532)	.001	13.4
Genotyping method	PCR‐RFLP	Allelic	0.853 (0.737–0.988)	.033	63.1
		Heterozygous	1.166 (0.992–1.370)	.062	54.6
		Homozygous	1.223 (0.861–1.737)	.260	38.5
		Dominant	1.187 (1.013–1.391)	.034	57.9
		Recessive	1.184 (0.866–1.619)	.289	28.1
	Taqman	Allelic	1.063 (0.749–1.509)	.732	60.4
		Heterozygous	1.040 (0.633–1.707)	.878	71.9
		Homozygous	1.037 (0.666–1.617)	.871	1.9
		Dominant	1.049 (0.690–1.596)	.823	63.7
		Recessive	1.131 (0.413–3.100)	.811	39.7
Smoking	Only smoking	Allelic	0.891 (0.628–1.265)	.520	66
		Heterozygous	1.154 (0.843–1.580)	.371	48.1
		Homozygous	0.951 (0.381–2.369)	.913	26.1
		Dominant	1.143 (0.806–1.622)	.452	60.4
		Recessive	0.967 (0.497–1.882)	.922	14.5
Tumor site	Oral cavity	Allelic	0.732 (0.602–0.891)	.002	59.7
		Heterozygous	1.471 (1.172–1.846)	.001	49.3
		Homozygous	1.460 (0.942–2.265)	.091	41.8
		Dominant	1.462 (1.208–1.769)	<.001	35.7
		Recessive	1.279 (0.773–2.116)	.338	59.1
	Pharynx	Allelic	0.828 (0.536–1.277)	.393	86.1
		Heterozygous	1.229 (0.813–1.857)	.329	77
		Homozygous	1.288 (0.381–4.359)	.684	75.7
		Dominant	1.235 (0.768–1.985)	.384	83.5
		Recessive	1.189 (0.424–3.330)	.742	67
	Larynx	Allelic	1.107 (0.790–1.551)	.556	0
		Heterozygous	0.844 (0.579–1.231)	.378	0
		Homozygous	1.400 (0.415–4.730)	.588	0
		Dominant	0.874 (0.606–1.262)	.472	0
		Recessive	1.514 (0.452–5.068)	.501	0

Abbreviations: CI, confidence interval; OR, odds ratio; PCR‐RFLP, Polymerase chain reaction‐restriction fragment length polymorphism.

#### 3.2.1 The association of XRCC1 Arg194Trp polymorphism with HNSCC risk based on different genetic models

Variants of *XRCC1* Arg194Trp polymorphism were associated with increased risk of HNSCC development based on different genetic models; the associations were significant under heterozygous and dominant genetic models (*p* values <.05).

**FIGURE 2:**
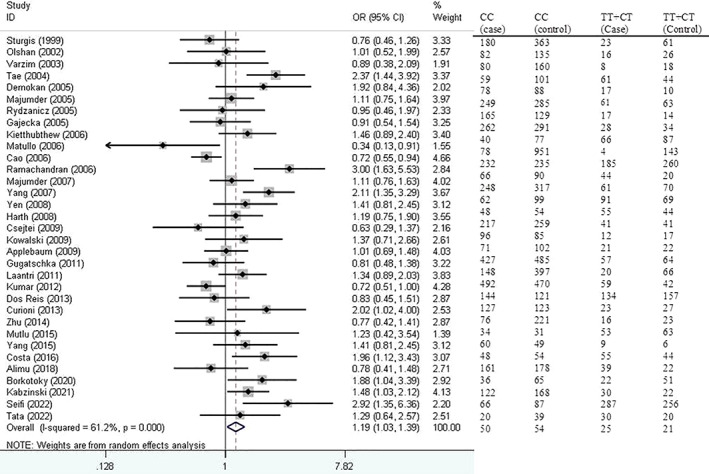
Forest plot for the association of *XRCC1* Arg194Trp polymorphism with head and neck squamous cell carcinoma (HNSCC) risk based on dominant model

Figure 2 shows forest plot for the association of *XRCC1* Arg194Trp polymorphism with HNSCC risk based on dominant model.

#### 3.2.2 The association of XRCC1 Arg194Trp polymorphism with HNSCC risk based on different ethnicities

There were not significant associations between *XRCC1* Arg194Trp polymorphism with HNSCC risk based on Caucasian or mixed ethnicity under different genetic models (*p* values >.05); the association was significant for Asian ethnicity under dominant genetic model so that the Trp/Trp + Arg/Trp variant was significantly associated with increased HNSCC risk compared to Arg/Arg variant.

**FIGURE 3:**
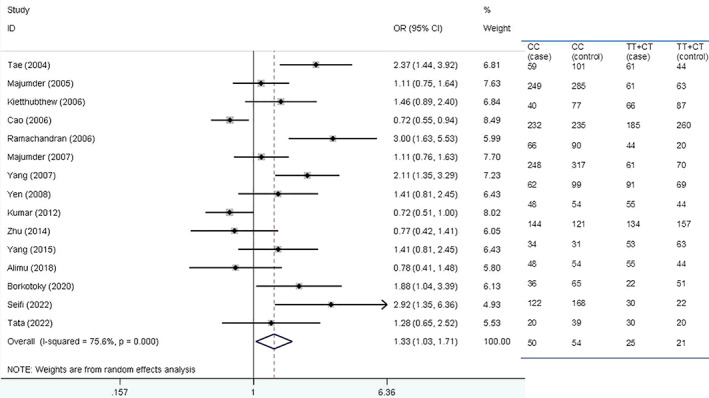
Forest plot for the association of *XRCC1* Arg194Trp polymorphism with head and neck squamous cell carcinoma (HNSCC) risk based on Asian ethnicity under dominant model

Figure 3 shows forest plot for the association of *XRCC1* Arg194Trp polymorphism with HNSCC risk based on Asian ethnicity under dominant model.

#### 3.2.3 The association of XRCC1 Arg194Trp polymorphism with HNSCC risk based on control source

There was significant association between *XRCC1* Arg194Trp polymorphism with HNSCC risk based on hospital‐based control source under different genetic model (*p* values <.05); variants of this polymorphism increased the HNSCC risk compared to corresponding reference variant.

There were not any significant associations between *XRCC1* Arg194Trp polymorphism with HNSCC risk based on healthy control source under different genetic models.

**FIGURE 4:**
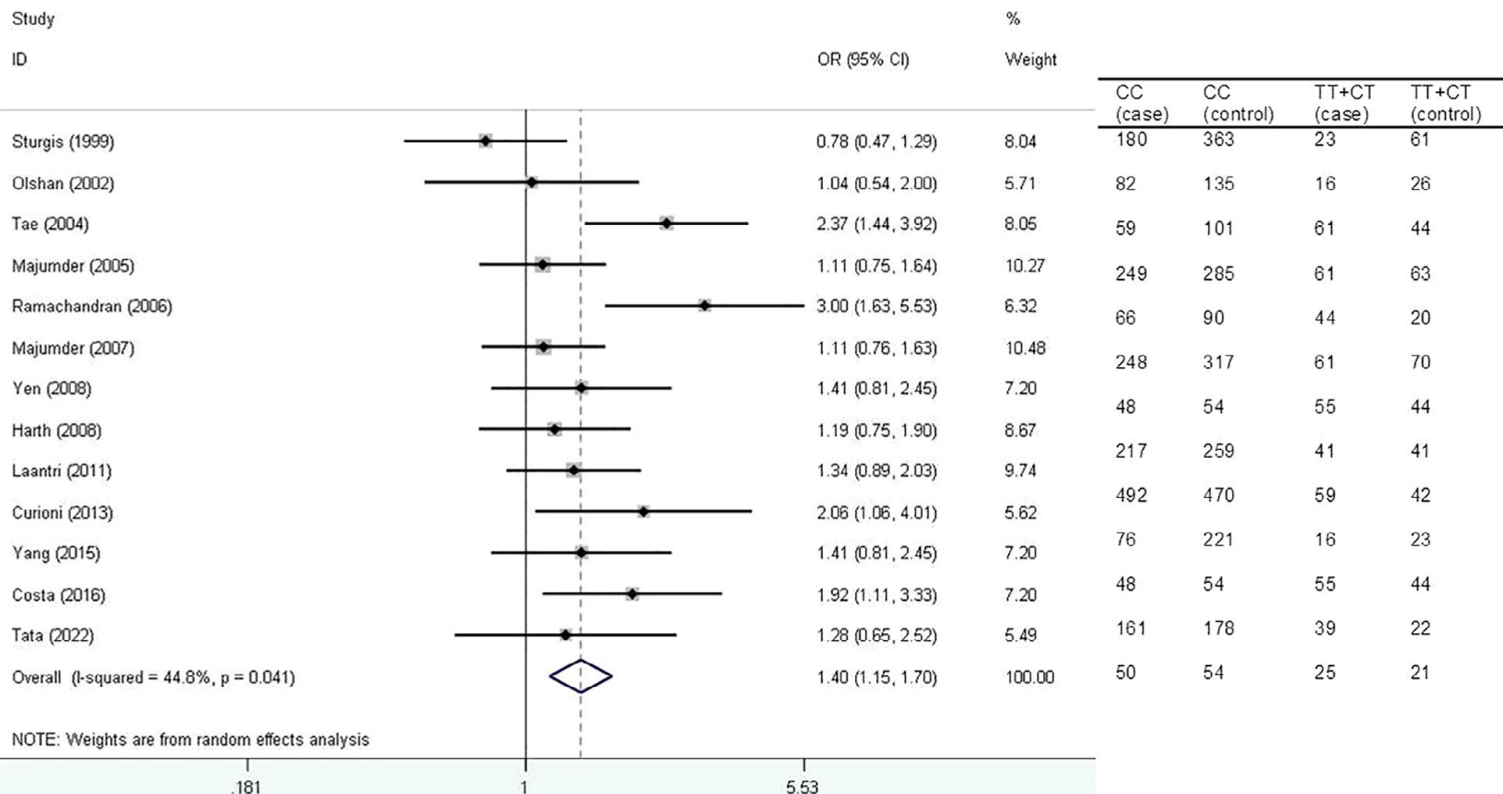
Forest plot for the association of *XRCC1* Arg194Trp polymorphism with head and neck squamous cell carcinoma (HNSCC) risk based on hospital‐based control source under dominant model

Figure 4 shows forest plot for the association of *XRCC1* Arg194Trp polymorphism with HNSCC risk based on hospital‐based control source under dominant model.

#### 3.2.4 The association of XRCC1 Arg194Trp polymorphism with HNSCC risk based on genotyping method

There were not any significant associations between *XRCC1* Arg194Trp polymorphism with HNSCC risk based on Taqman genotyping method under different genetic models (*p* values >.05).

There was significant association between *XRCC1* Arg194Trp polymorphism with HNSCC risk based on Polymerase Chain Reaction‐Restriction Fragment Length Polymorphism (PCR‐RFLP) genotyping method under dominant genetic model (*p* values <.05).

**FIGURE 5:**
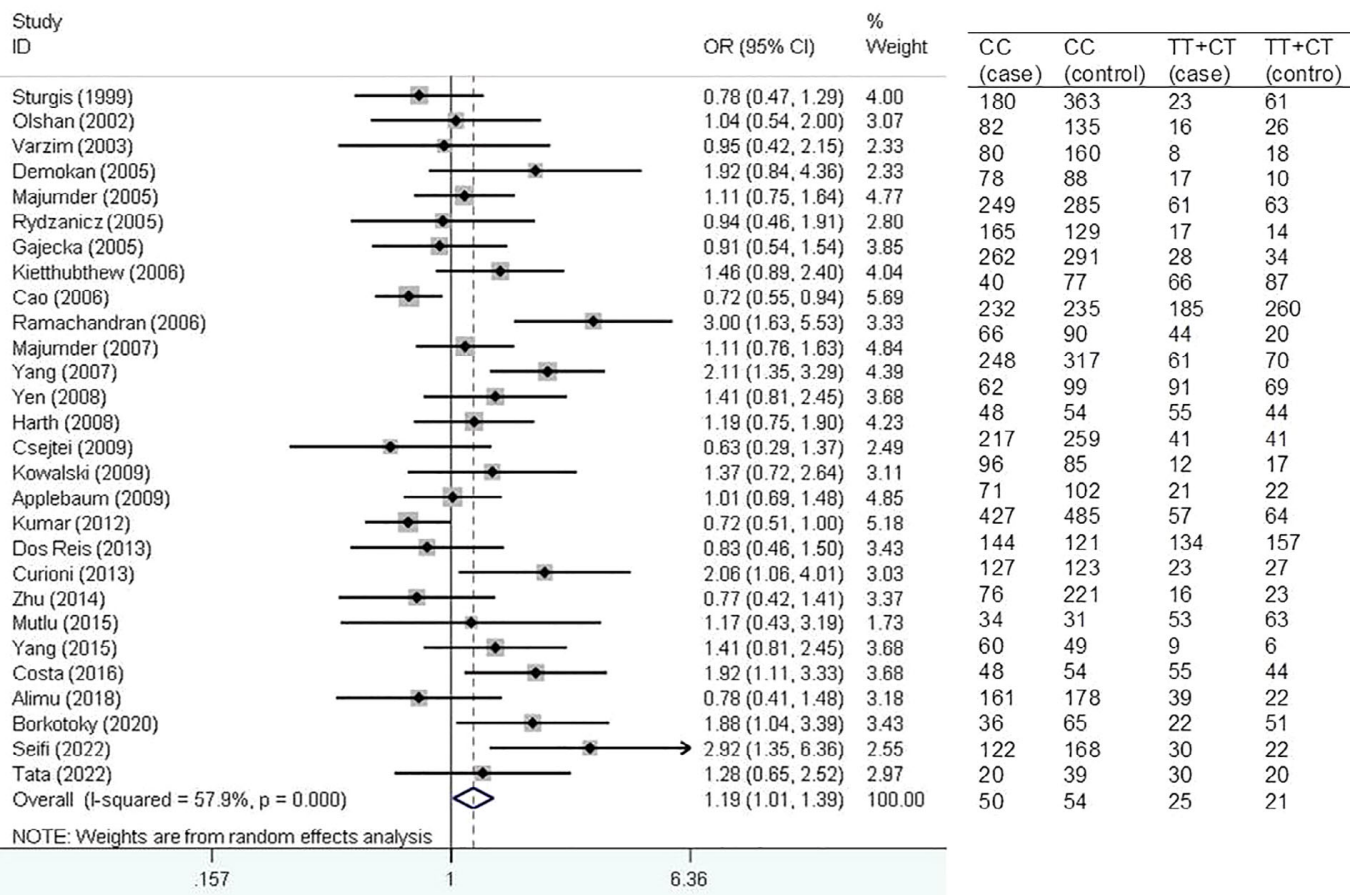
Forest plot for the association of *XRCC1* Arg194Trp polymorphism with head and neck squamous cell carcinoma (HNSCC) risk based on Polymerase Chain Reaction‐Restriction Fragment Length Polymorphism (PCR‐RFLP) genotyping method under dominant model

Figure 5 shows forest plot for the association of *XRCC1* Arg194Trp polymorphism with HNSCC risk based on PCR‐RFLP genotyping method under dominant model.

#### 3.2.5 The association of XRCC1 Arg194Trp polymorphism with HNSCC risk based on smoking participants

There were not any significant associations between *XRCC1* Arg194Trp polymorphism with HNSCC risk based on only smoking participants under different genetic models (*p* value >.001).

#### 3.2.6 The association of XRCC1 Arg194Trp polymorphism with HNSCC risk based on tumor site

There was significant association between *XRCC1* Arg194Trp polymorphism with HNSCC risk based on oral cavity tumor site under heterozygous and dominant models (*p* values <.05); the Arg/Trp + Trp/Trp (CT + TT) genotypes were significantly associated with increased risk of HNSCC development compared to Arg/Arg (CC) genotype (dominant model); also, the Arg/Trp variant significantly increased the HNSCC risk compared to Arg/Arg (heterozygous model); the associations were not significant under other genetic models (*p* value >.05).

There were not significant associations between *XRCC1* Arg194Trp polymorphism with HNSCC risk based on pharyngeal or laryngeal tumor sites under different genetic models (*p* values >.001).

**FIGURE 6:**
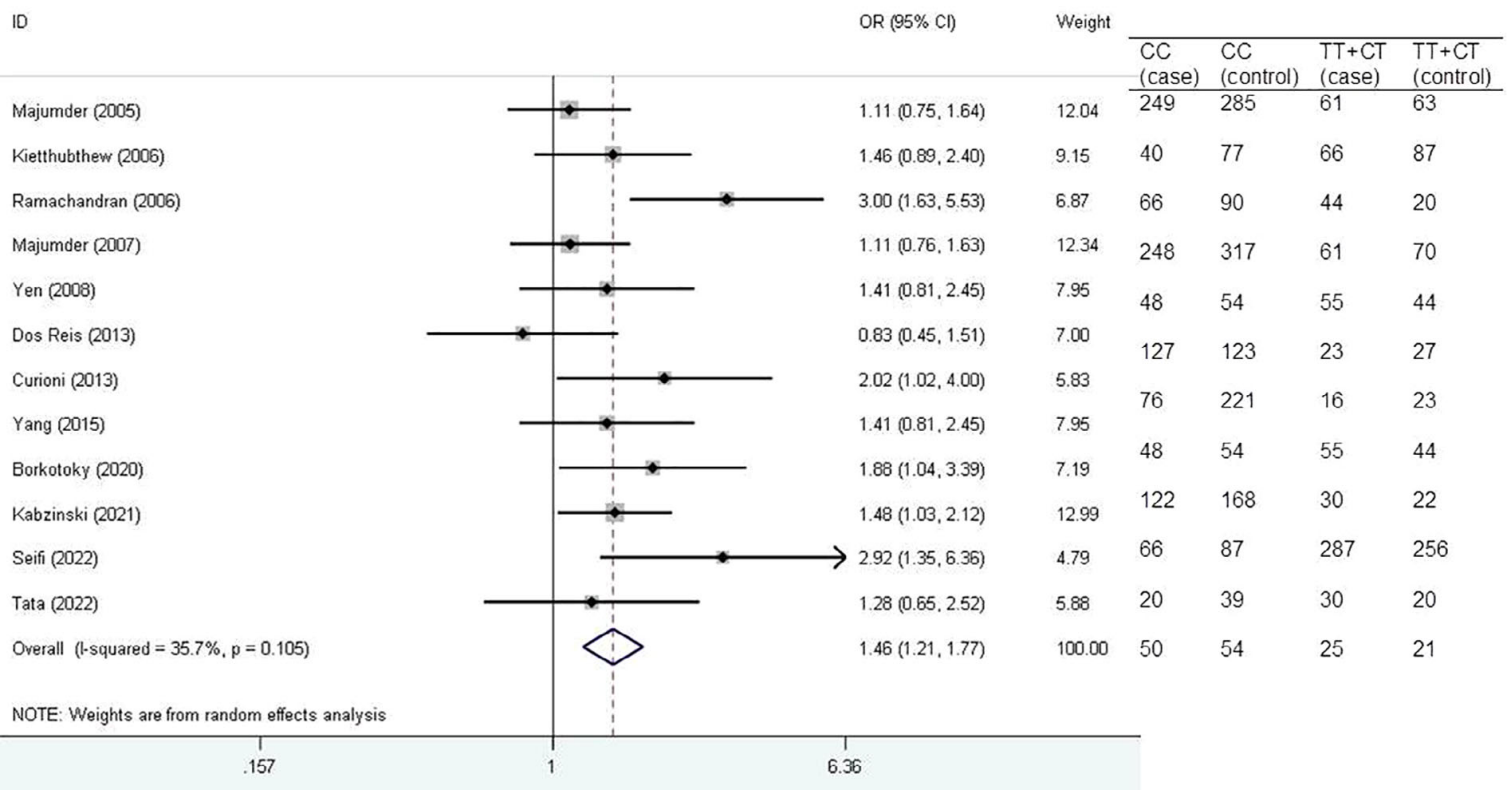
forest plot for the association of *XRCC1* Arg194Trp polymorphism with head and neck squamous cell carcinoma (HNSCC) risk based on oral cavity tumor site under dominant model

Figure 6 shows forest plot for the association of *XRCC1* Arg194Trp polymorphism with HNSCC risk based on oral cavity tumor site under dominant model.

## 4 DISCUSSION

This meta‐analysis showed that variants of *XRCC1* Arg194Trp polymorphism significantly increased the risk of HNSCC development under heterozygous and dominant genetic models; of course, it should be noted that although variants of this polymorphism was associated with increased risk of HNSCC development under homozygous and recessive genetic models, the association was not significant; subgroup analyses showed that there were significant associations between variants of this polymorphism and HNSCC risk based on Asian ethnicity under dominant model, hospital control source under different genetic models, PCR‐RFLP genotyping method under dominant model and oral cavity tumor site under heterozygous and dominant models.

The presence of associations between *XRCC1* Arg194Trp polymorphism and HNSCC risk under heterozygous and dominant genetic models shows that this polymorphism may play a role in individual differences in susceptibility to HNSCCs. Therefore, one of the ways to prevent head and neck cancer can be to identify genetically susceptible people (e.g., with unfavorable polymorphic variants of *XRCC1* Arg194Trp polymorphism) and undergo continuous and regular monitoring to prevent them from developing cancer and if head and neck cancer occurs, they can be diagnosed in the early stages. The insignificant results under homozygous and recessive genetic models show that these genetic models fail to identify relatively small effects of this single nucleotide polymorphism on HNSCC development against a complex background of biological factors or large‐scale population‐based studies are needed to reveal such an effect under these genetic models.

In Hu et al.,^39^ Huang et al.,^40^ and Feng et al.^41^ and meta‐analyses on the *XRCC1* Arg194Trp polymorphism and risk of cancer, this polymorphism was identified as a biomarker of cancer risk. In Hu et al. meta‐analysis, under dominant genetic model, the Trp/Trp + Arg/Trp genotypes was significantly associated with decreased cancer risk compared to Arg/Arg genotype (OR = 0.89 [95% CI: 0.81–0.98]) for all tumor types (breast, lung, etc.); subgroup analysis in head and neck cancers showed similar results (OR = 0.85 [95%CI: 0.59–1.23])^39^; their results are inconsistent with the results of present meta‐analysis; the reason for this inconsistency may be due to the very small number of available studies on HNSCC in their meta‐analysis compared to a much larger number of the same studies in the present meta‐analysis. Huang et al. observed in their meta‐analysis that *XRCC1* Arg194Trp polymorphism is a cancer risk factor among Chinese population so that a significantly increased risk was found under recessive model (OR = 1.31; 95%CI: 1.13–1.53); in the subgroup analysis, this association was observed for lung and esophageal cancers; among the head and neck cancers, only nasopharyngeal carcinoma was present in their stratification which had no significant association with this polymorphism^40^; their general results on all type of cancers are consistent with the results of present meta‐analysis except for genetic model. In Feng et al. met‐analysis, a significant increased risk was found under recessive, homozygous and additive models; their results are consistent with the results of present meta‐analysis except for genetic models.^41^

In Flores‐Obando et al. meta‐analysis, there was a significant association between *XRCC1* Arg194Trp polymorphism with head and neck cancer risk^2^ which is consistent with the results of the present meta‐analysis; of course, in their meta‐analysis, the increased OR (OR = 1.69, 95% CI: 1.10–2.58) was observed under the homozygous model; in our meta‐analysis the significant association was observed under heterozygous and dominant genetic models; in their meta‐analysis, a significant increase in HNSCC risk was observed for Asian ethnicity which is consistent with the results of the present meta‐analysis. In both meta‐analyses, no significant association was found for Caucasians ethnicity under heterozygous, homozygous and dominant models. In their meta‐analysis, a significantly increased risk was observed for oral cancers; this is consistent with the results of the present meta‐analysis, although in their meta‐analysis, a significant association was obtained under heterozygous model; in the present meta‐analysis, this association was significant under heterozygous and dominant model.

In the three meta‐analyses conducted by Lou et al.,^1^ Wu et al.,^42^ and Zhou et al.,^43^ there were not significant association between *XRCC1* Arg194Trp polymorphism and the HNSCC risk under different genetic models; these findings are inconsistent with results of present meta‐analysis; the reason for these inconsistencies can be attributed to the smaller number of studies and the smaller number of cases and controls in their meta‐analysis. In Lou et al. and Wu et al. meta‐analyses, stratification analyses based on ethnicity and genotyping method; these findings are again inconsistent with results of present meta‐analysis. Variants of this polymorphism was significantly associated with a decreased risk of oral cavity cancer under recessive genetic model in Lou et al. meta‐analysis; the direction of results was inconsistent between our meta‐analysis and Lou et al. meta‐analysis in the field of oral cancers. Variants of this polymorphism was significantly associated with an increased risk of oral cavity cancer under the allelic, heterozygote, and dominant models in Wu et al. meta‐analysis; these findings of Wu et al. meta‐analysis are consistent with results of present meta‐analysis. In Lou et al. meta‐analysis,^1^ subgroup analysis for smoking showed significantly increased risk under homozygous model but in the present meta‐analysis no such association was found. In Zhou et al. meta‐analysis, stratification by ethnicity showed significant association in Asian ethnicity under heterozygous and recessive models^43^; their finding is consistent with the results of present meta‐analysis except for genetic model.

In Zhou et al. meta‐analysis, oral cancer susceptibility was not associated with *XRCC1* Arg194Trp polymorphisms, although there was significant increase in the risk of oral cancer in Asian ethnicity under allelic, homozygous, and dominant models.^44^ In contrast, there was significant association between this polymorphism with oral cavity cancer susceptibility under heterozygous and dominant models in the present meta‐analysis; the reason for the discrepancy could be related to a much larger number of cases and controls in the present meta‐analysis.

In Mozaffari et al. and Zhang et al. meta‐analyses on the association of *XRCC1* Arg194Trp with oral cancer risk, there were significant increased associations between this polymorphism and oral cancer risk under allelic, heterozygote, and recessive models in Mozaffari et al. meta‐analysis and under dominant model in Zhang et al. met‐analysis.^3^, ^45^ These results are consistent with the result of the present meta‐analysis. Subgroup analysis according to ethnicity in Zhang et al. meta‐analysis showed that this polymorphism was associated with significantly increased risk of oral cancer in Asians ethnicity under allelic, homozygous, and dominant genetic models.

In Lin et al. and Deng et al. meta‐analyses, there were no significant associations between *XRCC1* Arg194Trp polymorphism and nasopharyngeal carcinoma under all genetic models.^4^, ^46^ In the present meta‐analysis, there was also no significant association between this polymorphism and pharyngeal carcinomas.

Table 3 summarizes the results of above‐mentioned meta‐analyses for ease of comparison.

**TABLE 3:** The results of existing meta‐analyses on the association of *XRCC1* Arg194Trp polymorphism with cancer risk

First author, year	Type of cancer	Results			Number of			Subgroup analysis with significant result	Comments
		Non‐significant	Significant		Study	Case	Control		
			Increased risk	Decreased risk					
Hu, 2005^39^	All cancer types	‐	‐	√	38	11 957	14 174	‐	Significant results under dominant genetic model
	Head and neck	‐	‐	√	4	723	1045	‐	
Huang, 2011^40^	All cancer types	‐	√	‐	34	9374	12 111	‐	Only Chinese people analyzed; significant results under recessive model
	Nasopharynx	√	‐	‐	3	790	1013	‐	
Feng, 2014^41^	All cancer types	‐	√	‐	201	59 227	81 587	‐	Significant results under recessive, homozygous and additive models
Flores‐Obando, 2010^2^	Head and neck	‐	√	‐	15	2330	3834	Asian ethnicity (increased risk)	Significant results under the homozygous model
	Oral cavity	‐	√	‐	5	724	818	‐	Significant results under heterozygous model
Lou, 2013^1^	Head and neck	√	‐	‐	22	4478	6873	Smoking (increased risk)	Significant results under homozygous model
	Oral cavity	‐	‐	√	6	915	1412	‐	Significant results under recessive model
Wu, 2014^42^	Head and neck	√	‐	‐	21	3771	6144	‐	‐
	Oral cavity	‐	√	‐	7	1225	1760	‐	Significant results under the allelic, heterozygote, and dominant models
Zhou, 2014^43^	Head and neck	√	‐	‐	20	3362	5796	Asian ethnicity (increased risk)	Significant results under heterozygous and recessive models
Zhou, 2009^44^	Oral cavity	√	‐	‐	8	1362	3130	Asian ethnicity (increased risk)	Significant results under allelic, homozygous and dominant models
Mozaffari, 2021^3^	Oral cavity	‐	√	‐	7	1067	1602	‐	Significant results under allelic, heterozygote, and recessive models
Zhang, 2013^45^	Oral cavity	‐	√	‐	6	828	1412	Asian ethnicity (increased risk)	Significant results under dominant model
Lin, 2018^4^	Nasopharynx	√	‐	‐	5	1428	1519	‐	‐
Deng, 2017^46^	Nasopharynx	√	‐	‐	4	877	1007	‐	Restricted to Chinese population
Present meta‐analysis	Head and neck	√	‐	‐	33	6012	8270	Ethnicity/smoking/genotyping method (all non‐significant)	‐
	Oral cavity	‐	√	‐	12	1913	2266		Significant result under dominant model

### 4.1 Limitations

Among the limitations of the present meta‐analysis was the lack of sufficient information in some studies about variables such as age and sex.

## 5 CONCLUSION

Variants of *XRCC1* Arg194Trp polymorphism were associated with increased risk of HNSCC development under different genetic models; the associations were significant under heterozygous and dominant genetic models. There were significant associations between variants of this polymorphism and HNSCC risk based on Asian ethnicity, hospital control source, PCR‐RFLP genotyping method and oral cavity tumor site. Variants of this polymorphism were not significantly associated with increased risk of HNSCC development under different genetic models although they were associated with borderline decreased risk of HNSCC development under recessive genetic model.

## AUTHOR CONTRIBUTIONS

**Nooshin Mohtasham:** Conceptualization (equal); supervision (equal); writing – review and editing (equal). **Khadijeh Najafi‐Ghobadi**: conception and design of the study (equal); data collection and analysis (equal); data interpretation and drafting the manuscript (equal); critical revision of the manuscript (equal). **Hamid Abbaszadeh**: conception and design of the study (equal); data collection and analysis (equal); data interpretation and drafting the manuscript (equal); critical revision of the manuscript (equal).

## CONFLICT OF INTEREST

The authors have stated explicitly that there are no conflicts of interest in connection with this article.

## ETHNIC STATEMENT

Not applicable.

## Data Availability

The data related to this study is within the text.
